# Association Between Possible Sarcopenia and Fracture Risk Assessment Tool (FRAX)-Estimated Fracture Risk Without Bone Mineral Density in Institutionalized Older Adults: A Cross-Sectional Study

**DOI:** 10.7759/cureus.103678

**Published:** 2026-02-15

**Authors:** Callista N Yubaitsa, Karina S Gani, Mitchel Mitchel, Erica Kholinne

**Affiliations:** 1 Surgery, Faculty of Medicine, Universitas Trisakti, Jakarta, IDN; 2 Orthopedics and Traumatology, Gatam Institute, Eka Hospital, Tangerang, IDN

**Keywords:** elderly, fracture, frax, handgrip strength, sarcopenia

## Abstract

Background

Musculoskeletal problems in the elderly pose a significant challenge for healthcare providers, as they constitute a vulnerable group. Fractures are often undetected, leading to a reduced quality of life due to increased disability, morbidity, and mortality. Therefore, early detection and intervention are essential to prevent fractures in the elderly. Sarcopenia is a contributing factor, as it affects postural instability, decreased balance, and increases the risk of falls. However, the association between possible sarcopenia and fracture risk in the elderly remains uncertain.

Methods

This analytical observational study used a cross-sectional design. This study was conducted in October 2025. A total of 96 older adults in Panti Sosial Tresna Werdha (PSTW) Budi Mulia 2 were selected using consecutive sampling. Fracture risk was assessed using the Fracture Risk Assessment Tool (FRAX) questionnaire. Possible sarcopenia status was measured using handgrip strength based on the Asian Working Group for Sarcopenia (AWGS) 2019 criteria. Data were analyzed using univariate and bivariate analysis with the Fisher-Freeman-Halton exact test, with a significance level of p<0.05.

Results

The mean age of the respondents was 69.7±7.5 years; 87 respondents were categorized as possible sarcopenia (93.5%), while six respondents (6.5%) had no possible sarcopenia. Fracture risk assessment showed that 75 respondents were in the low-risk category (80.6%). Statistical analysis yielded a p-value of 0.791, indicating no significant relationship between possible sarcopenia and fracture risk in older adults at PSTW Budi Mulia 2.

Conclusion

Among institutionalized older adults, most participants were classified as having possible sarcopenia, while fracture risk assessed using the FRAX tool was predominantly low. No significant association was observed between possible sarcopenia defined by handgrip strength and FRAX-estimated fracture risk.

## Introduction

As age increases, the likelihood that an individual will experience physical, psychological, spiritual, economic, and social problems also rises [1]. Older adults, defined by the World Health Organization (WHO) as individuals aged 60 years and above, represent a rapidly growing population worldwide. In 2019, the global population aged 60 years and older reached one billion and is projected to increase to 2.1 billion by 2050 [2]. This rapid demographic transition, particularly in developing countries, presents significant challenges in providing adequate health and social services for this vulnerable age group [2,3].

Fractures constitute a significant health issue among the elderly, as they are associated with mobility limitations, disability, reduced quality of life, and increased mortality. The risk of fractures increases with advancing age due to multiple factors, including low bone mineral density (BMD), impaired balance, muscle weakness, and comorbid conditions such as osteoporosis and diabetes [4]. Fractures in older adults may occur following high-energy trauma or, more commonly, low-energy events such as falls. Data from the 2018 Indonesian Basic Health Research (Riskesdas) reported a fracture prevalence of 5.5% in Indonesia [5].

Accurate assessment of fracture risk in older adults requires tools that integrate multiple clinical risk factors. The Fracture Risk Assessment Tool (FRAX), developed by the WHO, is a widely used and validated instrument that estimates the 10-year probability of major osteoporotic and hip fractures [6].

Sarcopenia, characterized by the progressive loss of muscle mass and strength, is another crucial factor contributing to fracture risk in older people. It leads to postural instability, impaired balance, and an increased risk of falls, which are the leading causes of fractures in this population [7]. The Asian Working Group for Sarcopenia (AWGS) 2019 recommends screening for possible sarcopenia in primary healthcare settings using muscle strength or physical performance measures. Handgrip strength measurement was employed in this study because it is a simple, rapid, and non-invasive method that reflects overall muscle function and functional capacity in older adults [8]. Globally, the prevalence of sarcopenia among older people is estimated to be approximately 10%, while in Indonesia it reaches 17.6% [8].

However, evidence regarding the direct relationship between possible sarcopenia and fracture risk remains inconclusive. While Gandham et al. reported a significant association between sarcopenia and increased fracture risk, Nguyen et al. found no significant association between sarcopenia alone and fracture occurrence [9,10]. Therefore, this study aims to evaluate the association between possible sarcopenia and fracture risk among older adults using FRAX and handgrip strength measurement, to support early risk identification and improve fracture prevention strategies in the elderly population.

## Materials and methods

This study was an analytical, observational, unique-centered cross-sectional analysis conducted at a single point in time to assess the relationship between possible sarcopenia (independent variable) and fracture risk (dependent variable) among older adults. The study was carried out at Panti Sosial Tresna Werdha (PSTW) Budi Mulia 2, an elderly care institution located in Cengkareng Barat Subdistrict, Cengkareng District, West Jakarta City, Special Capital Region of Jakarta. The target population consisted of all individuals aged ≥60 years, while the accessible population comprised older adults aged ≥60 years residing at PSTW Budi Mulia 2, totaling 105 individuals. This study received approval from the Research Ethics Commission of Fakultas Kedokteran Universitas Trisakti (approval number: 023/KER/FK/09/2025) before the study began, and written informed consent was obtained from the participants. 

Study subjects were selected based on inclusion and exclusion criteria. The inclusion criteria comprised male and female older adults aged ≥60 years who were able to follow instructions and undergo examinations. The exclusion criteria included respondents with a history of major fractures who were unable to do the test, those with severe or terminal illnesses that could affect functional status such as severe neurological disorders or severe chronic diseases, respondents who were currently using or had a history of long-term high-dose corticosteroid use, individuals unable to undergo handgrip strength measurement, and those with cognitive or psychiatric disorders.

The data collected were primary data obtained through interviews and physical examinations. Fracture risk over 10 years was assessed using FRAX without BMD measurement, which was accessed online. The information collected included age, sex, body weight, height, history of previous fractures, parental history of fractures, smoking status, glucocorticoid use, history of rheumatoid arthritis, secondary osteoporosis, and alcohol consumption of ≥3 units per day, converted according to National Health Service (NHS) standards. FRAX questions were administered directly to respondents or, when necessary, with assistance from caregivers, using yes-or-no responses, and the final results were expressed as a percentage fracture risk [11]. FRAX was selected because it is a widely validated and internationally recognized instrument that integrates multiple clinical risk factors to estimate the 10-year probability of major osteoporotic and hip fractures. It can be applied with or without BMD measurement, making it particularly suitable for settings where densitometry is not readily available. Due to its practicality, accessibility, and applicability in primary healthcare and resource-limited environments, FRAX was considered an appropriate and standardized method for fracture risk assessment in this institutionalized elderly population [6].

Possible sarcopenia assessment was conducted based on AWGS 2019 recommendations [12]. In this study, possible sarcopenia was defined and assessed solely based on muscle strength, specifically handgrip strength, due to limitations in direct muscle mass measurement. Handgrip strength was measured using a handgrip dynamometer with the subject standing and the arm fully extended. This approach was selected in accordance with AWGS 2019 recommendations, which state that in primary healthcare settings, sarcopenia may be estimated based on muscle strength or physical performance as an initial, practical, and cost-effective screening method. Low muscle strength was defined as a handgrip strength of <28 kg in men and <18 kg in women [12].

In addition, calf circumference was measured with a measuring tape, with cut-off values of <34 cm for males and <33 cm for females, in accordance with AWGS 2019 recommendations. In this study, calf circumference measurements were used as supporting data to assess peripheral muscle status. They were not used as the basis for diagnosing sarcopenia, as direct muscle mass measurement was not performed. Calf circumference was used as an additional measure to assess consistency between muscle strength and peripheral muscle size.

Data were analyzed using IBM SPSS Statistics for Windows, Version 28.0 (IBM Corp., Armonk, New York, United States). Univariate analysis was used to describe respondent characteristics and the distribution of each study variable, expressed as frequencies and percentages. Bivariate analysis was conducted to assess the relationship between possible sarcopenia and fracture risk using the Fisher-Freeman-Halton exact test with a 95% confidence level. A p-value of <0.05 was considered statistically significant, indicating a significant association between possible sarcopenia and fracture risk among older adults.

## Results

The mean age of the respondents was 69.7±7.5 years, with 50 men and 43 women. Based on fracture risk assessment using FRAX without BMD, the majority of respondents were categorized as having a low fracture risk, accounting for 75 individuals (80.6%). Regarding possible sarcopenia diagnosis, based on muscle strength assessment using a handgrip dynamometer, most respondents, 87 individuals (93.5%), met the criteria for possible sarcopenia. Calf circumference measurement showed that 48 respondents (51.6%) had calf circumference below the normal cut-off. The detailed information is provided in Table 1.

**Table 1:** Distributions of respondents' characteristics FRAX: Fracture Risk Assessment Tool

Characteristics	Total N (%)	Male N (%)	Female N (%)
Fracture risk (FRAX)			
Low fracture risk	75 (80.6)	48 (64)	27 (36)
Moderate fracture risk	14 (15.1)	1 (7.1)	13 (92.9)
High fracture risk	4 (4.3)	1 (25)	3 (75)
Sarcopenia			
Possible sarcopenia	87 (93.5)	45 (51.7)	42 (48.3)
No possible sarcopenia	6 (6.5)	5 (83.3)	1 (16.7)
Calf circumference			
Cut-off	45 (48.4)	27 (60)	18 (40)
Below the cut-off value	48 (51.6)	22 (45.8)	26 (54.2)

Based on the Fisher-Freeman-Halton exact test, there was no statistically significant association between possible sarcopenia and fracture risk (p=0.791). This test was applied because more than 66.7% of the cells had expected counts of fewer than five, violating the chi-squared test's assumptions. Therefore, the Fisher-Freeman-Halton exact test was used as an alternative for cells with low frequencies (Table 2).

**Table 2:** The relationship between possible sarcopenia and fracture risk *Fisher-Freeman-Halton exact test (p>0.05) FRAX: Fracture Risk Assessment Tool

Possible sarcopenia	Fracture risk (FRAX)						Total		P-value
	Low		Moderate		High				
	n	%	n	%	n	%	n	%	
Yes	69	74.2%	14	15.1%	4	4.3%	87	93.5%	0.791*
No	6	6.5%	0	0%	0	0%	6	6.5%	

The results showed that the majority of respondents with possible sarcopenia were classified as having a low fracture risk, totalling 69 individuals (74.2%). In contrast, all respondents without possible sarcopenia were also categorized as having a low fracture risk, with no individuals in the moderate or high fracture risk categories. These findings indicate that fracture risk among older adults was not significantly associated with possible sarcopenia. Consequently, it can be concluded that there is no significant relationship between possible sarcopenia and fracture risk in the elderly population.

## Discussion

Multiple factors influence fracture risk in older adults. Based on the results of this study conducted at PSTW Budi Mulia 2, West Jakarta, involving 93 older adults aged ≥60 years, the mean age of respondents was 69.7 years with a standard deviation of 7.5 years, indicating that most participants were classified as young-old to old-old adults. Regarding sex distribution, 50 male respondents (53.8%) and 43 female respondents (46.2%) were included, reflecting a relatively balanced distribution of older adults in the institution. However, when FRAX scores were examined in detail, women tended to have higher FRAX scores than men [13]. Additionally, this study found that 13 of the 14 respondents with moderate fracture risk and three of the six respondents with high fracture risk were women. These findings are consistent with the literature, which indicates that women, particularly after menopause, experience an increased fracture risk due to decreased estrogen levels that lead to increased bone resorption and reduced bone formation, resulting in bone mass loss and a higher incidence of osteoporosis [14]. This condition can be explained by postmenopausal hormonal changes, specifically a significant decline in estradiol levels accompanied by increased follicle-stimulating hormone (FSH) and luteinizing hormone (LH), which have been shown to play a role in the progressive reduction of bone mass and the increased occurrence of osteopenia and osteoporosis [15]. Furthermore, in FRAX calculations, female sex is epidemiologically associated with a higher fracture risk compared to males [16].

Regarding sarcopenia, diagnosis based on the AWGS 2019 criteria, using muscle strength assessment with a handgrip dynamometer, showed that most respondents (87 individuals, 93.5%) met the criteria for possible sarcopenia, while only six individuals (6.5%) were classified as possibly not sarcopenic. This proportion is consistent with the concept that older adults residing in institutional settings, such as social care homes, are at a high risk of muscle mass and strength decline due to aging, limited physical activity, and comorbidities, as described in previous studies on the etiology and risk factors of sarcopenia, including the study by Bravo-José et al. That study reported that more than 40% of long-term care residents experienced sarcopenia, with risk increasing with advanced age, female sex, low body mass index, and higher levels of functional dependence, collectively reflecting high vulnerability to muscle mass and strength loss in institutionalized elderly populations [17]. Muscle strength, as measured by handgrip strength, is closely associated with overall muscle function and functional capacity in older adults [8].

In addition, this study measured calf circumference as supporting data to assess peripheral muscle status, using cut-off values of <34 cm for males and <33 cm for females in accordance with AWGS 2019 recommendations. Unlike body mass index, which has limited ability to represent muscle mass due to its inability to differentiate between fat mass and muscle mass and its susceptibility to central fat distribution, calf circumference is considered more sensitive for detecting muscle mass reduction in older adults [18]. Although calf circumference was not used as a diagnostic criterion for sarcopenia, most respondents had measurements near the lower limit of normal, consistent with the high proportion of possible sarcopenia and reflecting reduced peripheral muscle mass among institutionalized older adults. Reduced peripheral muscle mass may contribute to skeletal fragility, increased fall risk, and fracture risk, particularly when combined with other factors such as advanced age and suboptimal nutritional status [19].

Bivariate statistical analysis using the Fisher-Freeman-Halton exact test yielded a p-value of 0.791 (p>0.05), indicating no statistically significant association between possible sarcopenia and fracture risk among older adults at PSTW Budi Mulia 2. These findings suggest that although the prevalence of possible sarcopenia was high, it was not statistically associated with low, moderate, or high fracture risk categories as calculated using FRAX without BMD. This suggests that fracture risk among respondents was likely more strongly influenced by a combination of other factors already incorporated in FRAX, such as age, body mass index, sex, fracture history, comorbidities, and other clinical risk factors, rendering the contribution of possible sarcopenia as a single variable less prominent in this analysis.

Several methodological factors may explain the absence of a significant association. For example, a study by Hars et al. comparing multiple diagnostic criteria for sarcopenia reported a significant association between sarcopenia and fracture risk when using the Baumgartner criteria, which define sarcopenia based on appendicular skeletal muscle mass (ASM) adjusted for height [20]. Similarly, a meta-analysis by Yeung et al. reported a positive association between sarcopenia and increased fracture risk; however, sarcopenia in that analysis was assessed using a combination of muscle mass, muscle strength, and physical performance, resulting in higher estimated fracture risk [21]. In contrast, sarcopenia in the present study was defined solely on the basis of handgrip strength, according to AWGS 2019 criteria, without the assessment of muscle mass or physical performance, thus reflecting only possible sarcopenia. Furthermore, FRAX was calculated without BMD values, meaning that fracture risk estimation relied entirely on clinical risk factors, which may not parallel muscle strength status.

The relatively small sample size and recruitment from a single social care institution may also have reduced the statistical power to detect differences, particularly given the uneven distribution of fracture risk and possible sarcopenia categories. Studies reporting significant associations between sarcopenia and fracture risk generally involve more diverse and community-dwelling elderly populations rather than institutionalized cohorts [20]. In addition, many of these studies employed prospective cohort designs that allowed for the direct assessment of fracture incidence during follow-up, enabling the more precise identification of temporal relationships between sarcopenia and fractures. Differences in study design and population characteristics may therefore explain why previous studies reported stronger associations than those observed in the present study [17].

Other studies, such as that by Lim et al., did report a significant association between sarcopenia and fractures, but only through the mechanism of fragile falls. Moreover, the study population consisted of post-injury older adults undergoing rehabilitation, in whom post-fracture immobilization may have exacerbated sarcopenia [22].

Overall, these findings highlight inconsistencies across existing studies and support the notion that possible sarcopenia is not the sole determinant of fracture risk but rather part of a complex interplay of risk factors, including osteoporosis, nutritional status, chronic comorbidities, and medication use [23]. This is consistent with findings by Harris et al., who reported that sarcopenia alone does not significantly increase fracture risk, particularly in the absence of low BMD, and that fracture risk increases primarily among individuals with low BMD regardless of sarcopenia status [24]. In addition, fracture risk in individuals with sarcopenia may be influenced by impaired physical performance, reduced gait speed, poor balance, and increased fall propensity, which directly contribute to fragility fractures [22]. The coexistence of sarcopenia and osteoporosis has been shown to confer a higher fracture risk than either condition alone. Nutritional deficiencies, particularly inadequate protein and vitamin D intake, as well as polypharmacy including corticosteroid use, may further exacerbate muscle weakness, bone fragility, and fall risk [20,22].

This study has several limitations. First, BMD was not measured, which may limit the comprehensiveness of fracture risk evaluation and the ability to assess osteosarcopenia. Second, additional variables that may influence fracture risk, such as physical activity level, nutritional status, and comorbidities, were not comprehensively assessed. Third, the cross-sectional design precludes the determination of temporal or causal relationships. Furthermore, the relatively small sample size and recruitment from a single social care institution may limit generalizability and reduce variability in clinical risk factors and FRAX scores. In addition, sarcopenia status was defined using handgrip strength alone, reflecting possible sarcopenia rather than a full diagnostic assessment, and the highly skewed distribution of sarcopenia status limited meaningful comparison between groups. Future studies should incorporate BMD assessment, broader clinical variables, prospective cohort designs, and larger multi-center samples to provide a more comprehensive evaluation.

## Conclusions

This study did not demonstrate a significant association between possible sarcopenia, as determined by handgrip strength, and fracture risk estimated using FRAX in institutionalized older adults. These findings indicate that possible sarcopenia alone may not serve as an independent predictor of fracture risk, underscoring the multifactorial etiology of fractures in the elderly population. The results also imply that fracture risk assessment and preventive interventions in institutionalized older adults should incorporate a comprehensive evaluation of multiple risk factors rather than concentrating exclusively on possible sarcopenia. Nonetheless, possible sarcopenia remains a clinically relevant condition that requires attention due to its contribution to functional impairment and diminished quality of life among older individuals.
